# In turkeys, unlike chickens, the non-structural NS1 protein does not play a significant role in the replication and tissue tropism of the H7N1 avian influenza virus

**DOI:** 10.1080/21505594.2024.2379371

**Published:** 2024-07-16

**Authors:** Maryna Kuryshko, Maria Landmann, Christine Luttermann, Reiner Ulrich, Elsayed M. Abdelwhab

**Affiliations:** a Institute of Molecular Virology and Cell Biology, Friedrich-Loeffler-Institut, Federal Research Institute for Animal Health, Greifswald-Insel Riems, Germany; b Institute of Veterinary Pathology, Faculty of Veterinary Medicine, Leipzig University, Leipzig, Germany; c Institute of Immunology, Friedrich-Loeffler-Institut, Federal Research Institute for Animal Health, Greifswald-Insel Riems, Germany

**Keywords:** Virulence determinants, avian influenza virus, turkeys, NS1, interferon, replication

## Abstract

The economic losses caused by high pathogenicity (HP) avian influenza viruses (AIV) in the poultry industry worldwide are enormous. Although chickens and turkeys are closely related Galliformes, turkeys are thought to be a bridging host for the adaptation of AIV from wild birds to poultry because of their high susceptibility to AIV infections. HPAIV evolve from low pathogenicity (LP) AIV after circulation in poultry through mutations in different viral proteins, including the non-structural protein (NS1), a major interferon (IFN) antagonist of AIV. At present, it is largely unknown whether the virulence determinants of HPAIV are the same in turkeys and chickens. Previously, we showed that mutations in the NS1 of HPAIV H7N1 significantly reduced viral replication in chickens *in* *vitro* and *in* *vivo*. Here, we investigated the effect of NS1 on the replication and virulence of HPAIV H7N1 in turkeys after inoculation with recombinant H7N1 carrying a naturally truncated wild-type NS1 (with 224 amino-acid “aa” in length) or an extended NS1 with 230-aa similar to the LP H7N1 ancestor. There were no significant differences in multiple-cycle viral replication or in the efficiency of NS1 in blocking IFN induction in the cell culture. Similarly, all viruses were highly virulent in turkeys and replicated at similar levels in various organs and swabs collected from the inoculated turkeys. These results suggest that NS1 does not play a role in the virulence or replication of HPAIV H7N1 in turkeys and further indicate that the genetic determinants of HPAIV differ in these two closely related galliform species.

## Introduction

Avian influenza viruses (AIV) belong to the family *Orthomyxoviridae* and the genus Influenza A virus (IAV). AIV are further subdivided into 16 HA and 9 NA subtypes according to variations in the surface glycoprotein, haemagglutinin (HA or H), and neuraminidase (NA or N). AIV infect a wide range of bird species. Wild birds are natural reservoir of AIV. Infection of wild birds with AIV is, with rare exceptions, asymptomatic, whereas in poultry, it can cause high mortality with huge economic impact. AIV usually have two types of pathogenicity: low pathogenicity (LP) and high pathogenicity (HP) forms [1]. HPAIV H5 and H7 subtypes evolve from LP ancestors by acquisition of point mutations in different proteins or by reassortment (swapping) of gene segments between two different AIVs infecting the same cell [1]. Although the acquisition of a polybasic cleavage site in HA is a major determinant of AIV pathogenicity [2], other genes contribute to the virulence and pathogenesis of HPAIV [3]. Compared to chickens, very little is known about the genetic determinants for replication and virulence of HPAIV in turkeys, the second most kept poultry species in Europe and the USA after chickens.

Non-structural protein 1 (NS1) contributes to virulence in chickens and ducks [4,5]. NS1 is encoded by the shortest segment of the AIV genome with a typical length of 230 amino acids (aa). NS1 is a multifunctional protein consisting of an RNA binding domain (RBD) linked by a short linker (LR) to an effector domain (ED) [6]. The RBD of the NS1 protein binds to different types of RNA, preventing the activation of host cell sensors for foreign viral RNAs. ED interacts with various host factors via multiple motifs to trigger different pathways of the innate immune response following AIV infection [7,8]. Variations in stop codons in the ED, resulting in the truncation of the carboxyl terminus (CTE), may lead to variations in the size of NS1. Truncation of the CTE of NS1 has been described to affect virus replication *in* *vitro* in a strain- and cell-dependent manner [7,8]. For example, elongation of the NS1 CTE reduced H7N1 replication in chicken fibroblasts [9] but did not affect H5N1 or H9N2 replication [10,11]. Conversely, shortening the NS1 CTE increased H5N8 replication in turkey cells, but did not affect virus replication in chicken, human or canine cells [12].

In 1999, the H7N1 LPAIV emerged in March and the HPAIV emerged in December in Italy. Several genetic changes accompanied this shift to HPAIV as described previously [13]. Truncation of 6-aa in the NS1 CTE and three mutations in the ED accompanied the evolution of Italian HPAIV H7N1. Previously, we showed that the extension of NS1 of the Italian HPAIV H7N1 (from 224-aa to 230-aa, similar to the LP precursor) reduced HPAIV replication and cell/tissue tropism in chickens [14]. Similarly, reduced virulence has been observed in chicken embryos [9]. Although turkeys were the most affected species among the 13 million dead birds during the Italian outbreak [15], little is known about the effect of NS1 CTE truncation on AIV virulence in turkeys. Here, we investigated the effect of NS1 length variation on virus replication *in vitro* as well as virulence and transmission in experimentally inoculated turkeys.

## Results

### Sequence analysis suggested that turkeys, unlike chickens, were infected with H7N1 AIV carrying variable NS1 of different lengths

**Figure 1.**
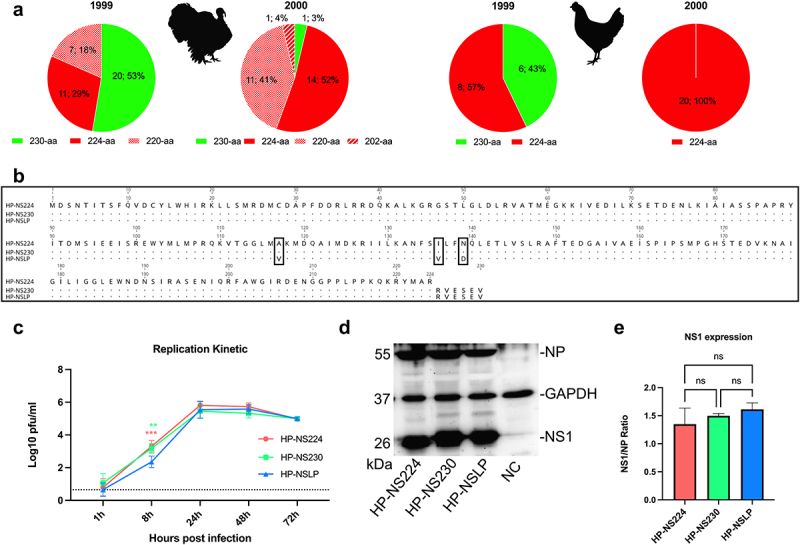
Sequence analysis and *in* *vitro* characterization of recombinant H7N1 viruses.

Note: (a) Sequence analysis of NS1 of H7N1 from turkeys (*n* = 65) and chickens (*n* = 28) in 1999–2000 deposited in GISAID (retrieval date 16-01-2024). Variations in NS1 length are shown as number and percentage of total sequences analysed in different years. Sequence analysis was performed using Geneious Prime. (b) Alignment and amino acid differences of the NS1 protein of the viruses used for this study. (c) Turkey embryonic kidney (TEK) cells were infected at a MOI 0.001 and virus titres were determined at 1, 8, 24, 48 and 72 h post infection (hpi) by plaque assay performed on MDCK II cells. Titres were calculated as pfu/ml and are presented as the mean and standard deviation of three independent experiments performed in duplicate. Data were analysed by one-way ANOVA with post hoc Tukey test. Asterisks indicate significant differences (*= *p* < 0.05, **= *p* < 0.01, ***= *p* < 0.001, ****= *p* < 0.0001); ns = no significant differences. (d) NS1 protein was detected after infection of TEK cells at a MOI of 0.1 for 24 h at 37°C. Detection was performed using rabbit polyclonal sera (D. Marc, INRAE, Nouzilly, France) and ECL substrate. The Western blot image was acquired using Quantity One software version 4.4 (Biorad, Germany). (e) Image J was used to calculate NS1 expression levels and data are presented as NS1/NP ratio.

We analysed the NS1 sequences of Italian H7N1 available in the GISAID on 16 January 2024 (*n* = 124) isolated from turkeys (*n* = 71), chickens (*n* = 35) and other birds (*n* = 18). As previously reported [13,14], all H7N1 LPAIV in 1999 contained full-length NS1 with 230-aa. Interestingly, almost all non-turkey isolates in 2000 contained a short NS1 of 224-aa in length. Conversely, turkey isolates in 2000 (*n* = 27) showed variations in NS1 length and possessed NS1 with 230-aa (*n* = 1/27, an LP), 224-aa (*n* = 14/27), 220-aa (*n* = 11/27) or 202-aa (*n* = 1/27) (Figure 1(a)). *These results suggest that, in contrast to chickens, there may be no positive selection or advantage for NS1 CTE variations in H7N1 isolated from turkeys*.

### Recombinant viruses and mutants

To assess the effect of NS1 length variation on the fitness of the Italian HPAIV H7N1, we used three recombinant viruses, generated in a previous study [14]. Each of these viruses contains seven gene segments (segments 1 to 7) of the Italian H7N1 HPAIV and variable NS segments (Figure 1(b)): HP-NS224 (containing NS from HP with NS1 of 224-aa length), HP-NS230 (containing NS from HP with extended CTE similar to LP NS1) and HP-NSLP (containing NS from LP virus with 230aa length and 3-aa substitutions in the ED: V117A, V136I and D139I). Prior to use, all viruses were sequenced and were found to have no undesirable mutations.

### NS1 did not significantly affect multi-cycle replication of HPAIV H7N1 in primary turkey cells

A previous study showed that NS1 CTE and ED affected the replication of an Italian H7N1 virus in chicken cells [9], while another study did not find any difference in the replication of LP H7N1 with similar NS1–230 or NS1–224 in chicken or duck cells [16]. Here, we investigated the influence of NS1 on HPAIV H7N1 replication in freshly prepared primary turkey embryo kidney (TEK) cells infected at a multiplicity of infection (MOI) of 0.001 for single cycle (8 hours post infection “hpi”) and multiple cycle replication (24, 48 and 72 hpi) (Figure 1(c)). The virus titre was determined by plaque assay in Madin-Derby canine kidney cells type II (MDCKII) cells. All viruses reached their maximum replication rates at 24 hpi. There were no significant differences in the virus titres at 24, 48 or 72 hpi. At 8 hpi, HP-NSLP had a significantly lower titre than HP-NS224 and HP-NS230 (*p* < 0.01). *These results indicate that NS1 did not significantly affect the multiple-cycle replication of HPAIV H7N1 in primary turkey cells.*

### Variations in the NS1 protein did not significantly affect NS1 expression levels in primary turkey cells

To assess the effect of changes in CTE or ED on the expression of NS1 in turkey cells, TEK cells were infected with HP-NS224, HP-NS230 or HP-NSLP at a MOI of 0.1 pfu for 24 h (Figure 1(d,e)). Cell lysates were prepared and protein expression was assessed by Western blot. GAPDH and H7N1 nucleoprotein (NP) were used as internal controls. As expected, the molecular mass of NS1 correlated with its length of the NS1 protein. *However, there were no significant differences in the expression levels of the NS1 variants in TEK cells under these experimental conditions.*

### All NS1 proteins were comparably efficient at blocking IFN-α and IFN-β mRNA induction in cell culture

**Figure 2.**
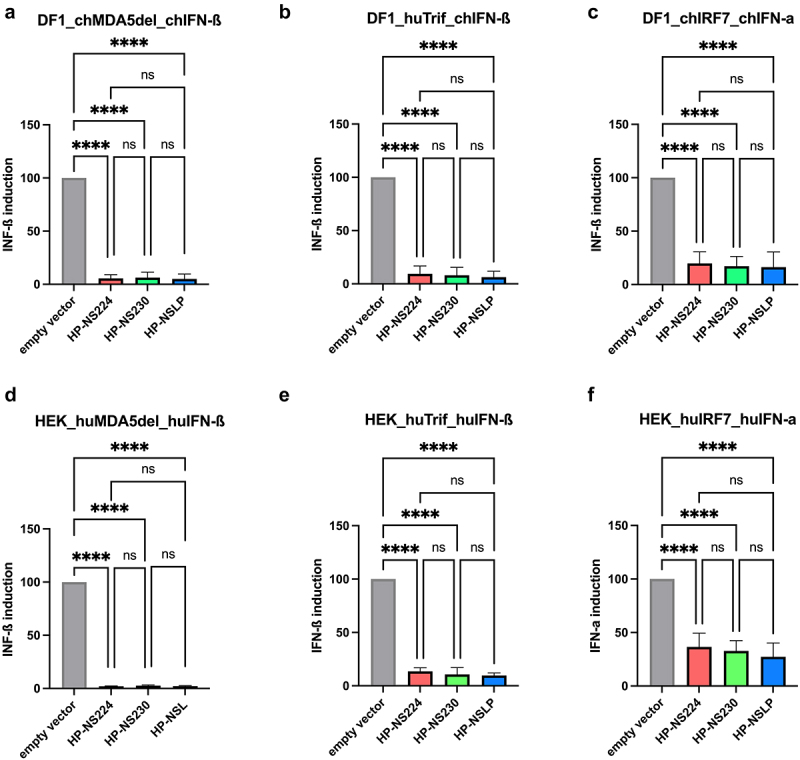
Inhibition of type I interferon induction in avian and human cell lines after transfection with H7N1 NS1.

Previous reports indicated that HPAIV NS1 was more efficient at inhibiting interferon (IFN)-induction of similar Italian HPAIV H7N1 in chicken fibroblast cells [9]. To investigate whether the length of NS1 affected the IFN antagonism of our H7N1 viruses, we used a firefly luciferase reporter assay after the transfection of human (HEK293T) and avian (DF1) cells (Figure 2). Attempts to transfect primary TEK cells were unsuccessful. The data were normalized to the signals induced in cells transfected with empty vectors. *All three NS1 pCAGGS expression plasmids were comparably efficient in blocking IFN-α and IFN-β mRNA induction in cells triggered by various avian and human factors such as MDA5, Trif and IRF7. Similarly, no significant differences were observed in the multi-cycle replication of the three viruses in human lung cells (A549) (Supplementary Figure S1)*.

The efficiency of NS1 to block IFN-α and -β mRNA induction was investigated in avian (DF1) (a-c) and human (HEK293T) (d-f) cells using a double reporter luciferase assay. Results are expressed as fold change of IFN-I promotor induction relative to the signal of the indicated trigger for the empty vector control. Asterisks indicate significant differences (*= *p* < 0.05, **= *p* < 0.01, ***= *p* < 0.001, ****= *p* < 0.0001). ch, chicken; hu, human; ns= no significant differences.

### Variations in NS1 did not significantly affect the high virulence or transmissibility of H7N1 AIV in turkeys

**Figure 3.**
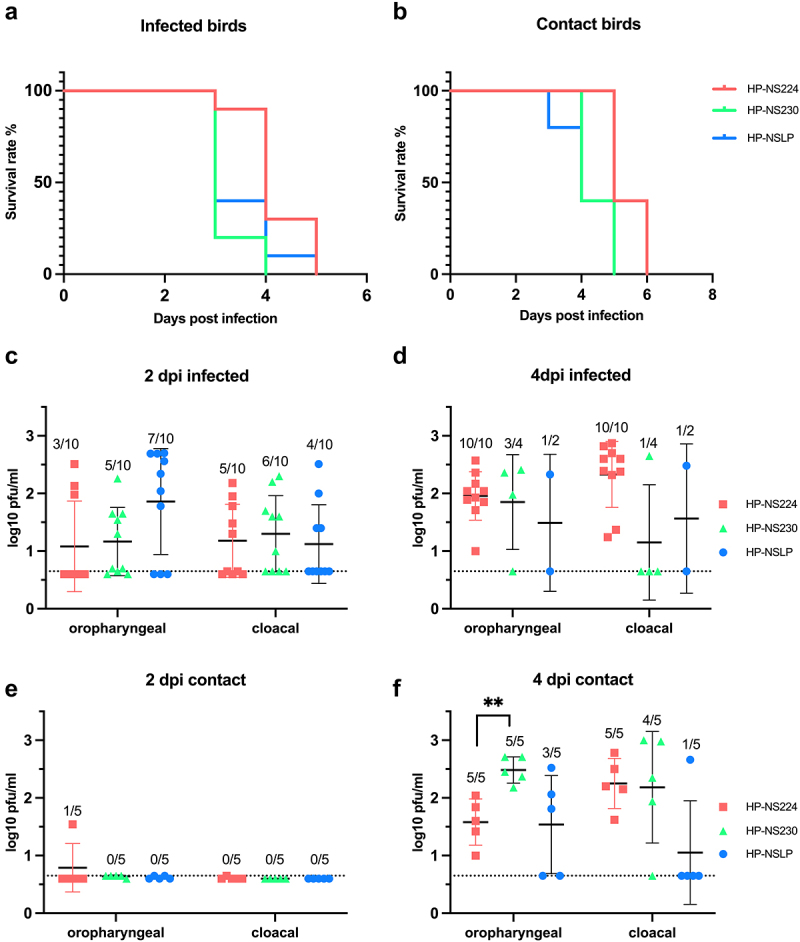
Effect of NS1 gene segment on survival and virus shedding after infection of turkeys with recombinant H7N1 viruses.

Previous studies have shown that NS1 mutations reduce the virulence of H5N1 HPAIV in chickens [5,17], whereas others have found no variations in the high virulence of H7N1 in chickens [14]. Furthermore, no data are available on the influence of NS1 on HPAIV transmission in turkeys. Therefore, we oculonasally inoculated 6-week-old turkeys (*n* = 10) with recombinant HPAIV H7N1 and 1-day post inoculation (dpi) naïve turkeys (*n* = 5) were added to each group to assess turkey-to-turkey transmission. The morbidity, mortality and clinical scores (ranging from 0 = avirulent to 3 = highly virulent) were recorded daily for 10 days (Table 1; Figure 3(a), B). Almost all birds developed clinical signs such as ruffled feathers, mild depression and neurological disorders starting 2 dpi and progressed moderately 3 dpi. All HP-NSLP-inoculated birds died 4 dpi and all HP-NS230 and HP-NS224-inoculated groups died at 5 dpi. The contact birds in all three groups started to show clinical signs at 3 dpi and were dead 5 dpi in the HP-NSLP and HP-NS230 groups and 6 dpi in the HP-NS224 group. No significant difference in the pathogenicity index (PI) was observed for any of the viruses, with mean clinical scores of 2.44, 2.42 and 2.48, respectively (Table 1; Figure 3(a,b)). *Taken together, NS1 did not significantly affect the high virulence or efficient transmission of the HPAIV H7N1 in turkeys*.

The survival rate is shown for both inoculated- (a) and contact-turkeys (b). Detection of viral load in swab samples from infected (c, d) and contact (e, f) birds. Oropharyngeal and cloacal swabs were collected 2- and 4-days post infection (dpi) and viral titres were measured by plaque assay using MDCKII cells. Asterisks indicate significant difference, **= *p* < 0.01. Dashed lines (C, D, E, F) indicate the predicted limit of detection of the plaque assay.

**Table 1.** Virulence of recombinant H7N1 viruses in inoculated and contact turkeys.

Viruses	Inoculated turkeys			Contact turkeys	
	Mortality	PI	MTD	Mortality	MTD
HP-NS224	10/10	2.44	4.2	5/5	5.4
HP-NS230	10/10	2.42	3.2	5/5	4.4
HP-NSLP	10/10	2.48	3.5	5/5	4.2

Ten turkeys were inoculated with 10^4.5^ pfu/ml of the indicated viruses. To assess bird-to-bird transmission, 5 naive birds were added to each inoculated group at 1-day post infection (dpi). Mortality refers to dead animals/total inoculated. Pathogenicity index (PI) from 0 (avirulent) to 3 (highly virulent) and mean time to death (MTD) per day after infection was calculated as the sum of daily arithmetic means divided by 10, the number of days observed. MTD in contact turkeys was calculated from the dpi of the primary inoculated birds.

### NS1 did not affect viral shedding in primary infected turkeys, but extension of NS1 CTE increased viral shedding in co-housed turkeys

Our previous experiment on chickens [14] showed that HP-NS1 extension reduced HPAIV H7N1 shedding and suggested a role for NS1 in virus transmission in chickens. Therefore, we measured infectious the viruses in cloacal and oropharyngeal swabs 2 and 4 dpi. The inoculated turkeys shed comparable amounts of the virus at 2 and 4 dpi, with no statistical differences between the groups. In sentinel turkeys, virus titres were comparable to those in primary-inoculated birds 4 dpi. The only significant difference was that HP-NS230 was shed in oropharyngeal swabs at significantly higher levels (~10 times higher) than that of HP-NS224 at 4 dpi (Figure 3(c–f)). *Collectively, these results suggested that NS1 did not significantly affect viral replication in primarily inoculated turkeys or transmission to co-housed turkeys.*

### NS1 did not greatly affect the histopathological lesions or tropism in inoculated turkeys

**Figure 4.**
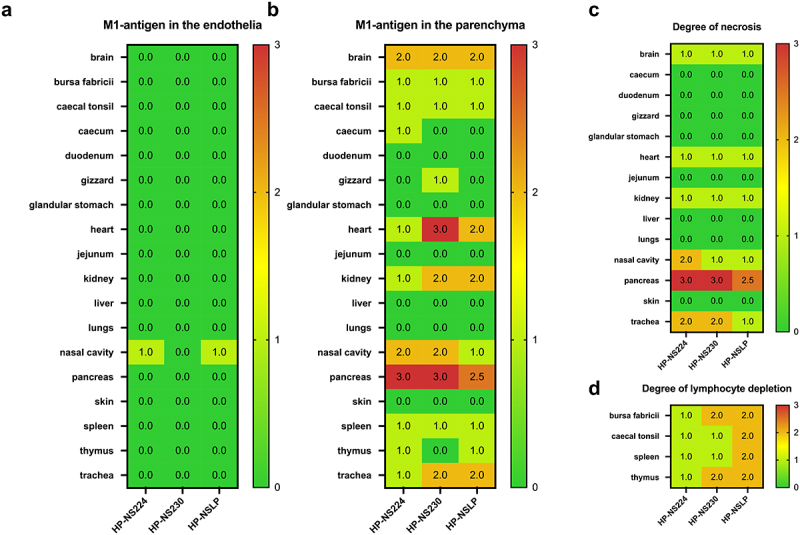
Distribution and pathological findings in H7N1-inoculated turkeys.

**Figure 5.**
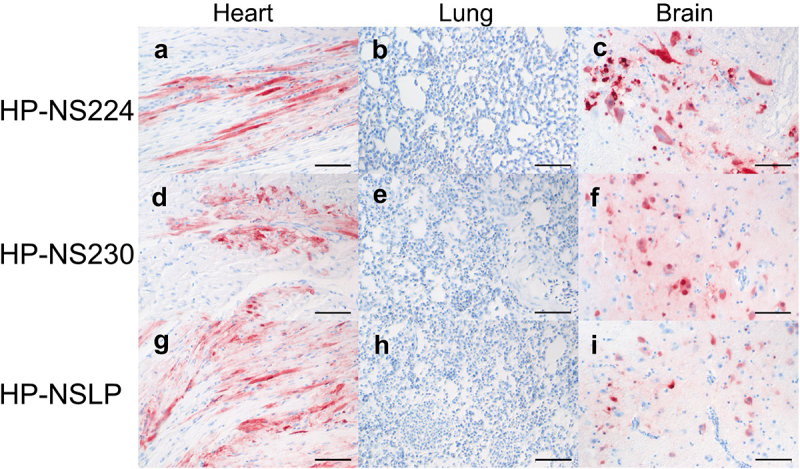
Detection of H7N1 matrix 1 protein in selected tissues of inoculated turkeys.

To assess the distribution of H7N1 matrix 1 (M1) antigen as an indicator of virus replication, organ samples collected at 4 dpi were subjected to immunohistochemical examination (Figure 4) similar to our previous chicken experiment [14]. No tropism to the blood vessel endothelium was observed in any of the turkeys, except for a few focal signals, mostly in the nasal cavities of HP-NS224 and HP-NSLP inoculated turkeys (Figure 4(a)). Conversely, semiquantitative assessment of M1 protein showed for all three viruses comparable, often multifocal or diffuse distribution of antigen in the parenchyma of different organs such as brain, heart, kidney, pancreas and trachea, but no or only rarely focal parenchymal antigen in the duodenum, proventriculus, liver, lung or skin (Figure 4(b) and 5). The distribution of M1 antigen in cardiomyocytes was more widespread in HP-NS230 than in the other two groups (Figure 4(b)). For all three viruses, similar levels of necrosis were also observed mainly in the brain, heart, kidney, nasal cavity, pancreas and trachea, ranging from mild to severe (Figure 4(c)). Depletion was observed in lymphoid organs in all turkeys examined, particularly in those inoculated with HP-NSLP, followed by HP-NS230 and HP-NS224 (Figure 4(d)). *These results indicate that variations in NS1 did not significantly affect virus distribution or histopathological changes in the major organs (e.g. brain, heart, kidney, spleen), which is consistent with clinical examination*.

Samples were collected from inoculated turkeys 4 dpi. M1 antigen distribution was assessed semiquantitatively in the endothelial cells (a) and parenchyma (b) of the indicated organs. The degree of necrosis (c) and lymphocyte depletion (d) was also assessed microscopically. The colour of the heatmap corresponds to the median of the semiquantitative scores: 0 = no, 1 = mild, 2 = moderate, 3 = severe lesion for necrosis and depletion, 0 = no, 1 = focal to oligofocal, 2 = multifocal, 3 = coalescing to diffuse antigen for parenchyma and 0 = no antigen, 1 = antigen in single blood vessels, 2 = antigen in multiple blood vessels, 3 = diffuse immunoreactivity for endothelial cells.

The maximum distribution of influenza A virus M1 protein 4 dpi in heart, lung and brain of turkeys inoculated with HP-NS224 (a, b, c), HP-NS230 (d, e, f) and HP-NSLP (g, h, i). Immunohistochemistry was done with the avidin-biotin-peroxidase-complex method, a primary monoclonal mouse antibody against influenza A virus matrix protein, 3-amino-9-ethylcarbazol (red-brown) as chromogen and haematoxylin (blue) as counterstain; Nomarski contrast; Bars = 50 µm.

### Co-localization of NS1 and M1 in the brain of H7N1 infected turkeys

**Figure 6.**
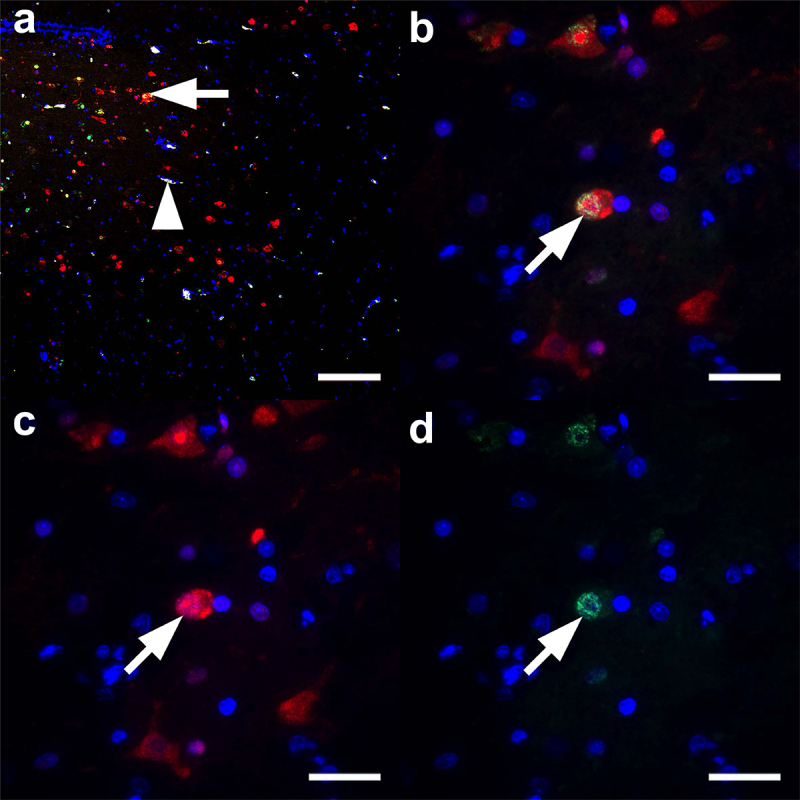
Co-localization of NS1 and M1 antigens in the brain tissues of H7N1 inoculated turkeys.

To date, NS1 as a non-structural protein is not considered to be part of the infectious virion and no data are available on the expression or distribution of NS1 in infected birds. As a proof of principle, we sought to identify NS1 in the brain of HP-NS224 infected turkeys at 4 dpi using immunofluorescence (Figure 6(a–d)). Interestingly, multiple foci of M1 and NS1 antigen expression were detected in the brain. Higher magnification showed simultaneous M1 and NS1 expression in neurons, which was clearly identifiable by their characteristic morphology (Figure 6(b)). NS1 expression was equally strong in the cytoplasm and nucleus (Figure 6(c)), whereas M1 expression tended to be stronger in the nucleus (Figure 6(d)). The NS1 signal was stronger and better discriminated from the autofluorescent background than the M1 signal in immunofluorescence experiments. Simultaneously processed heart, spleen and lung tissues showed overwhelming autofluorescence due to the high amounts of erythrocytes, preventing reliable analysis of these tissues by immunofluorescence (data not shown). These results indicate that, similar to the M1 antigen, NS1 expression is stable in the brain of H7N1 inoculated turkeys at 4 dpi and can be used for *in* *vivo* experiments (e.g. to study interactions with host factors *in* *vivo*).

Immunofluorescence was used to proof simultaneous expression of influenza A virus non-structural antigen 1 (NS1) and matrix-1 protein (M1). The brain of a HP-NS224-infected turkey 4 dpi was immunolabelled for NS1 in red (c), M1 in green (d), both with nuclei in blue. In the merged images (a-b) co-localization of NS1 and M1 is shown in yellow and nuclei in blue. (a) The merged low magnification image of the cerebrum displays a periventricular focus (ependymal nuclei lined up like a ribbon in the upper left corner) of NS1 and M1 expression, often within polygonal cells, resembling neurons, visible as yellow double-labelling (arrow) in the merged image. The arrowhead points to a vessel exhibiting intense, white, false positive autofluorescence. (b-c) A higher magnification of a group of characteristic neurons displays an equal intensity of NS1 in the cytoplasm and nucleus, as compared to a more nuclear expression of M1 (a-d) Immunofluorescence for M1 with AlexaFlour 488-coupled secondary antibody, NS1 with Alexa-456-coupled secondary antibody and nuclei with 4,’6-diamidino-2-phenylindole. (a) Scale bar = 100 µm; (b-d) scale bars = 20 µm.

## Discussion

Turkeys and chickens are Galliformes and highly susceptible to AIV infection [18]. Because of their higher susceptibility to AIV infection than chickens, particularly to H7 viruses [19,20], turkeys play an important role in the adaptation of AIVs of wild bird origin to poultry [21,22]. In contrast to ducks, chickens and turkeys typically die after HPAIV infection. Therefore, it has been suggested that virulence determinants and virus-host interactions in AIV-infected turkeys are likely similar to those in chickens. However, we have recently shown that virulence determinants located in the HA of two different H7 viruses are different in chickens and turkeys [23,24]. No data are available on the role of NS1 in AIV fitness in turkeys. A few studies have shown that mutations in the NS1 of H7Nx, particularly in the ED or CTE, affect virus fitness in chicken embryos [9,11] chickens [16,25–29] and ducks [16,30]. Understanding the genetic determinants of adaptation and virulence of HPAIV in turkeys will help to assess the role of turkeys as an intermediate host for the transmission of AIV from wild birds to poultry and the mechanism for the evolution of HPAIV from LP ancestors.

In the current *in* *vitro* experiments, we found no significant differences in multiple-cycle viral replication or NS1 expression in turkey cells. Soubies et al. [16] found that the 6-aa truncation associated with the transition of the Italian LPAIV H7N1 to the HPAIV had no effect on LP virus replication in ducks or chickens *in* *vitro* and *in* *vivo*. Similarly, all three NS1s were equally effective in blocking type I IFN mRNA induction in chicken and human cell lines, consistent with the findings of Soubies et al. [16] who described comparable levels of type I IFN mRNA induced by similar H7N1 LP carrying NS230-aa or NS224-aa in chicken and duck cells. In contrast Keiner et al. [9] found that prolongation of the CTE of HPAIV H7N1 NS1 reduced the efficiency of blocking IFN mRNA induction using a different cell culture and methodology than that used in the current study. In our previous chicken experiment using the same three viruses, we found that NS1 elongation reduced viral replication in the tissues of infected chickens [14]. Conversely, in turkeys we did not observe significant variation in the distribution of the H7N1 in different organs, further ruling out a role for NS1 as a virulence determinant in turkeys [24], in contrast to chickens or chicken embryos [9,24]. It is not clear whether a similar variation in virulence determinants in chickens and turkeys can be seen for other HPAIVs (e.g. H5Nx). Previous studies have shown that chickens and turkeys differ in susceptibility, pathobiology and transmission following infection with H5Nx viruses [31,32]. However, data on the genetic determinants and host factors that may contribute to this variation are lacking and need to be further investigated.

Currently, it is widely accepted that NS1 is a non-structural protein that is only expressed in cells after infection and is not part of the virion. Anti-NS1 antibodies have been detected in the sera of birds experimentally infected with different AIV subtypes, suggesting that NS1 antibodies could be used for diagnostic purposes [33–35]. However, to date, no studies have reported the detection of NS1 antigen specifically in the brains of turkeys. Our pilot study showed that the NS1 antigen can be detected in the turkey brain, similar to and overlapping with M1, indicating that the virus is replicating in neurons. This will be useful in subsequent experiments to further improve our understanding of the potential role of NS1 *in vivo*, for example, in neurovirulence or blocking the immune response in the turkey brain.

In conclusion, we found that turkeys can be infected with AIV carrying different NS1 lengths and mutations without significant effects on multiple-cycle replication, and NS1 expression *in* *vitro*, without affecting the high virulence, efficient transmission and tissue distribution of HPAIV H7N1 in turkeys. These results are in contrast to our previous findings in chickens, where NS1 significantly affected viral replication *in* *vitro* and *in* *vivo*. Our results further suggest that the pathogenesis and genetic markers for adaptation of AIV are different in chickens and turkeys, although they are closely related galliform species. Further studies should be conducted to understand the mechanism underlying the variable effect of NS1 on virus fitness in chickens and turkeys.

## Material and methods

### Sequence analysis

The NS1 sequences of H7N1 isolated from turkeys and chickens in Italy in 1999 and 2000 were retrieved from GISAID on 16 January 2024. Sequences with double entries and identical amino acid sequences were further edited. Alignment was performed using Geneious Prime® Software (Version 2021.0.1) and the MAFFT package.

### Cells and recombinant viruses

Primary turkey embryonic kidney cells (TEK) were prepared from the kidneys of 21-day-old turkey embryos [24,36]. Human embryonic kidney 293T (HEK-293T), human lung adenocarcinoma A549 cells, Madin-Darby canine kidney type II (MDCKII) and chicken fibroblast (DF1) cell lines were obtained from the Cell Bank of Friedrich-Loeffler-Institut (FLI). For this study, previously generated recombinant HPAIV H7N1 were used [14]: A/chicken/Italy/445/1999 virus carrying the HP NS1 (HP-NS224), the LP NS1 from A/chicken/Italy/473/1999 virus (HP-NSLP) and the elongated HP NS1 (HP-NS230) [14]. Virus stocks were prepared in embryonated chicken eggs (ECE) obtained from specific-pathogen-free chickens (VALO BioMedia GmbH, Germany). The whole genome sequence of the three viruses was determined as previously done [14].

### Plasmids

pCAGGS plasmids carrying the NS inserts of HP-NS224, HP-NS230, and HP-NSLP were generated in this study. The NS segment was amplified using the Omniscript RT Kit (Qiagen, Germany) and Phusion® High-Fidelity DNA Polymerase (New England Biolabs, USA). PCR products were purified on a 1% agarose gel and extracted using the GeneJET Extraction Kit (Thermo Fisher Scientific, Germany). Competent E. coli XL1-blue strain was transformed and plasmids were isolated from bacterial cultures using the Plasmid Midi Kit (Qiagen, Germany). Sequencing of the three NS1 plasmids was performed by Eurofins (Germany).

### Luciferase reporter assay

To determine the efficiency of NS1 in inhibiting the IFN-I pathway, we conducted a luciferase reporter assay as previously described [12]. Briefly, HEK293T and DF1 cells in 6 well plates were transfected with a plasmid DNA mixture containing 0.5 μg of a Firefly Luciferase (FFL) expressing reporter plasmid (firefly luciferase reporter plasmids with human or chicken IFN-I promotors:pIFN-ß-Pro-FFL, pIFNa-Pro-FFL), 0.005 μg of pCMV-RL (normalization), 0.2 μg human or chicken pIRF7 or 0.5 μg human or chicken pMDA5-delta or human pTrif as a trigger expression plasmid and 0.5 μg of pCAGGS plasmid with one of the NS1 coding sequences or an empty vector as a control. Lipofectamine™ 2000 (Thermo Fisher) was used for transfection according to the manufacturer recommendations. At 24 h post-transfection, cell lysates were harvested and luciferase activity was measured using the Dual-Luciferase® Reporter Assay System (Promega, USA) according to the manufacturer’s instructions. Firefly and Renilla activities were measured using a TriStar^2^ S LB 942 Modular Multimode Microplate Reader (Berthold, Germany). The assay was performed in three independent experiments and the results were expressed as normalized means and standard deviations.

### Western blot

TEK cells, 80% confluence, were infected with viruses (MOI 0.1) for 1 h, washed twice with phosphate-buffered saline (PBS) and overlaid with Ham’s F12/IMDM supplemented with 0.2% bovine serum albumin (BSA, MP Biomedicals, USA). Cells were harvested at 24 h post infection (hpi). Samples were centrifuged at 10,000 rpm for 5 min, washed with 0.5 ml PBS, centrifuged at 10,000 rpm for 10 minutes, resuspended in 50 µl of PBS and 50 µl of Laemmli sample buffer 2×, for SDS-PAGE (Serva, Germany), boiled at 99°C for 10 min and stored at −20°C until further use. Samples were run on 12% SDS-PAGE gel at 200 V for 47 min, blotted with BioRad Turbo Blotter, blocked with 5% milk overnight. They were stained with rabbit anti-NS1 polyclonal antibody (kindly provided by Daniel Marc, INRAE, Nouzilly, France), rabbit anti-NP polyclonal antibody and GAPDH as a cell normalization control (Abcam, United Kingdom). Blots were developed using the Biorad VersaDoc system and quantified using Image J software.

### Replication kinetics

The replication rate of the recombinant viruses was assayed by infecting TEK cells and A549 at an MOI of 0.001 for 1 h. The cells were washed twice with PBS and overlaid with Ham’s F12/IMDM medium supplemented with BSA. Plates containing cells were incubated at 37°C in 5% CO2 for 1, 8, 24, 48 and 72 hours. Prior to titration by plaque assay, harvested cells were stored at −80°C.

### Plaque assay

The plaque assay was used for virus titration and virus titres were expressed as plaque-forming units per mL (PFU/mL). Ten-fold serial dilutions of each virus were incubated on MDCKII cells in 6-well plates for one hour at 37°C and 5% CO2, washed twice with PBS, 0.9% Bacto Agar/MEM mixture supplemented with 0.2% BSA (MP Biomedicals, USA) and incubated for 72 h at 37°C and 5% CO2. After incubation, the plates were fixed with 0.1% crystal violet in 10% formaldehyde solution. Viral titres were determined by counting the number of plaques under a microscope.

### Turkey experiment: Ethical approval

Animal experiments were performed in the Biosafety Level 3 (BSL3) facility of the FLI in accordance with the German Animal Welfare Act after approval by the authorized Ethics Committee of the State Office for Agriculture, Food Safety and Fisheries of Mecklenburg-Western Pomerania (LALLF M-V) under registration number 7221.3-1.1-051-12. ARRIVE guidelines were followed. A total of 45 six-week-old turkeys were purchased from a farm in Mecklenburg- Western Pomerania. Each recombinant virus was used to oculonasally infect ten birds per group at 10^4.5^ PFU/bird. After 24 h, five naive birds were added to each group. All birds were observed daily for clinical signs (depression, respiratory distress, diarrhoea, cyanosis of the comb, wattles or shanks, facial oedema and neurological signs) over a 10-day observation period. Clinical scoring followed the standard protocol [37] where healthy birds were scored (0), sick birds with one of the clinical signs were scored (1), severely sick birds with two or more signs were scored (2) and dead birds were scored (3). Moribund birds that were unable to eat or drink were humanely euthanized by inhalation of isoflurane (CP-Pharma, Germany), exsanguinated and scored as dead on the next observation day. The pathogenicity index (PI) was calculated as the sum of the daily arithmetic means of all infected birds divided by 10 (the number of observation days), with a final range of 0 (avirulent) to 3 (highly virulent).

### Virus shedding

Oropharyngeal and cloacal swabs were collected from all birds at 2 and 4 dpi. Swabs were stored at −80°C in 1.5 ml Dulbecco’s Modified Eagle Medium (DMEM) containing 1.05 mg enrofloxacin (Baytril, Bayer AG, Germany), 0.525 mg lincomycin (Mediserv Nord, Germany), 0.105 mg gentamycin (Genta, CP-Pharma, Germany) in sterile safe-lock Eppendorf tubes, previously mixed by vortexing for 30 s. The amount of infectious virus in plaque-forming units was determined by titration of the swab samples using the plaque assay described above.

### Histopathology and immunohistochemistry

For histopathology and immunohistochemistry, organ samples were fixed in 4% neutral buffered formaldehyde for >7 days, processed, embedded in paraffin and sectioned at 2–4 μm. For histopathology, slides were stained with haematoxylin and eosin. Immunohistochemistry was performed using the avidin-biotin-peroxidase complex method (Vectastain PK 6100; Vector Laboratories, Burlingame, CA, USA) with citric buffer (pH 6.0), a primary mouse monoclonal antibody against influenza A virus matrix 1 protein (M1, ATCC clone M1Hb-64, 1:100), a secondary biotinylated goat anti-mouse IgG (BA-9200, Vector Laboratories, Newark, USA, 1:200), 3-amino-9-ethylcarbazol as chromogen, and haematoxylin counterstain as described [38,39]. Mouse IgG (NBP1–97019-5 mg, Novus Biologicals USA, CO, USA) was used as an isotype control instead of the primary antibody and validated archival tissues were used as a positive control.

Scoring of histopathological lesions and viral antigen distribution was performed as previously described [40]. Briefly, necrosis or necrotizing inflammation and lymphoid depletion were each scored as follows 0 = no, 1 = mild, 2 = moderate, 3 = severe lesion, and viral antigen distribution was scored as follows: 0 = no, 1 = focal to oligofocal, 2 = multifocal, 3 = coalescing to diffuse antigen for parenchymal cells and 0 = no antigen, 1 = antigen in single blood vessels, 2 = antigen in multiple blood vessels, 3 = diffuse immunoreactivity for endothelial cells.

### Immunofluorescence double-staining

An immunofluorescence double-staining experiment was performed for proof-of-principle of influenza A virus matrix 1 protein (M1) and non-structural protein 1 (NS1) co-expression in the brain of an HP-NS224-infected turkey (same animal as shown in Figure 5 (a–c)), as well as a H4/H5-infected chicken, and a non-infected chicken from previous studies as positive and negative controls, respectively. Briefly, 2–4 µm slides of the formalin-fixed paraffin-embedded specimen were deparaffinized, and sequentially incubated in 0.5% H2O2 in methanol for 30 min for inhibition of endogenous peroxidase, in citric acid buffer (pH 6,0) at 96°C for 25 min for unmasking of antigens, in 0.1% Triton X-100 in Tris buffered saline (TBS) for 15 min for permeabilization, and SuperBlock^TM^ blocking buffer (Thermo Fisher Scientific, USA) for 30 min for blocking excess binding sites. Afterwards, the slides were incubated with either single or mixed primary antibodies against M1 (monoclonal mouse anti-M1 antibody (ATCC, clone: M1Hb-64, 1:10) and NS1 (polyclonal anti-rabbit, 1:1000) at 4°C overnight, followed by incubation with AlexaFluor 488-conjugated donkey anti-mouse IgG (1:500, Dianova) and AlexaFluor 568-conjugated goat anti-rabbit IgG (1:500, Abcam) secondary antibodies at room temperature for 1 h. After further washing steps, sections were counterstained using 4,’6-diamidino-2-phenylindole (DAPI, 1:300, Invitrogen) and mounted with glycerol-gelatin aqueous slide mounting medium (Sigma Aldrich). The labelled sections were analysed using a motorized Axioplan 2 Imaging fluorescence microscope (Carl Zeiss Microscopy Deutschland GmbH, Oberkochen) equipped with 25×/0.8 Plan-Neofluar water-immersion, 40×/1,2 Apochromat water-immersion, and 63×/1,2 Apochromat water-immersion objectives, an HBO 50 mercury-vapour short-arc lamp, AHF F31–000 (excitation 350/50, emission 460/50, for DAPI), AHF F41-054HQ (excitation 480/30, emission 527/30 HQ, for AF488), and AHF F41-007HQ (excitation 545/30, emission 610/75 HQ, for AF568) filter sets, and a monochrome 12 megapixel Axiocam 712 mono R2 CMOS camera. Images were acquired using a semi-automated multi exposure protocol within the ZEN 3.8 software.

### Statistic

Statistical analyses were performed using Graph Pad Prism, Version 10.1.1. One-way ANOVA was used to analyse viral titres, expression levels of NS1 and inhibition of IFN induction. Kruskal–Wallis tests and Mann-Whitney-Wilcoxon with Benjamini-Hochberg correction were used for clinical scoring. Survival analysis was performed using the log-rank (Mantel-Cox) test.

## Supplementary Material

Supplementary Figure S1.tiff

## Data Availability

The authors confirm that the data supporting the findings of this study are openly available in Zenodo at https://zenodo.org/records/11263714?token=eyJhbGciOiJIUzUxMiJ9.eyJpZCI6IjA3N2ZjOTI2LWMzZjgtNDc2NS1hNDAwLWQ2NTBiMmQ1MGY2ZiIsImRhdGEiOnt9LCJyYW5kb20iOiJkZTY0MTBhNjI1ZDNhZWZlZTE5NTRiNWY1NDdmNTVlMiJ9.KSM1L_hOVagodhV79z-YkGrRhCmnHAQALlIZwrNB4MpXEHC0qXzC11274D-udgAFGt8bPqOfQLm3FLeT_A9qIA

## Declaration

A preprint version of this manuscript is available online at: Preferential selection and contribution of non-structural protein 1 (NS1) to the efficient transmission of the panzootic avian influenza H5N8 2.3.4.4 clades A and B viruses in chickens and ducks | bioRxiv.
