# Supplementary figures and images for: In turkeys, unlike chickens, the non-structural NS1 protein does not play a significant role in the replication and tissue tropism of the H7N1 avian influenza virus
Source: Virulence. 2024 Jul 16;15(1):2379371. doi: 10.1080/21505594.2024.2379371 (PMC11259080; doi:10.1080/21505594.2024.2379371)

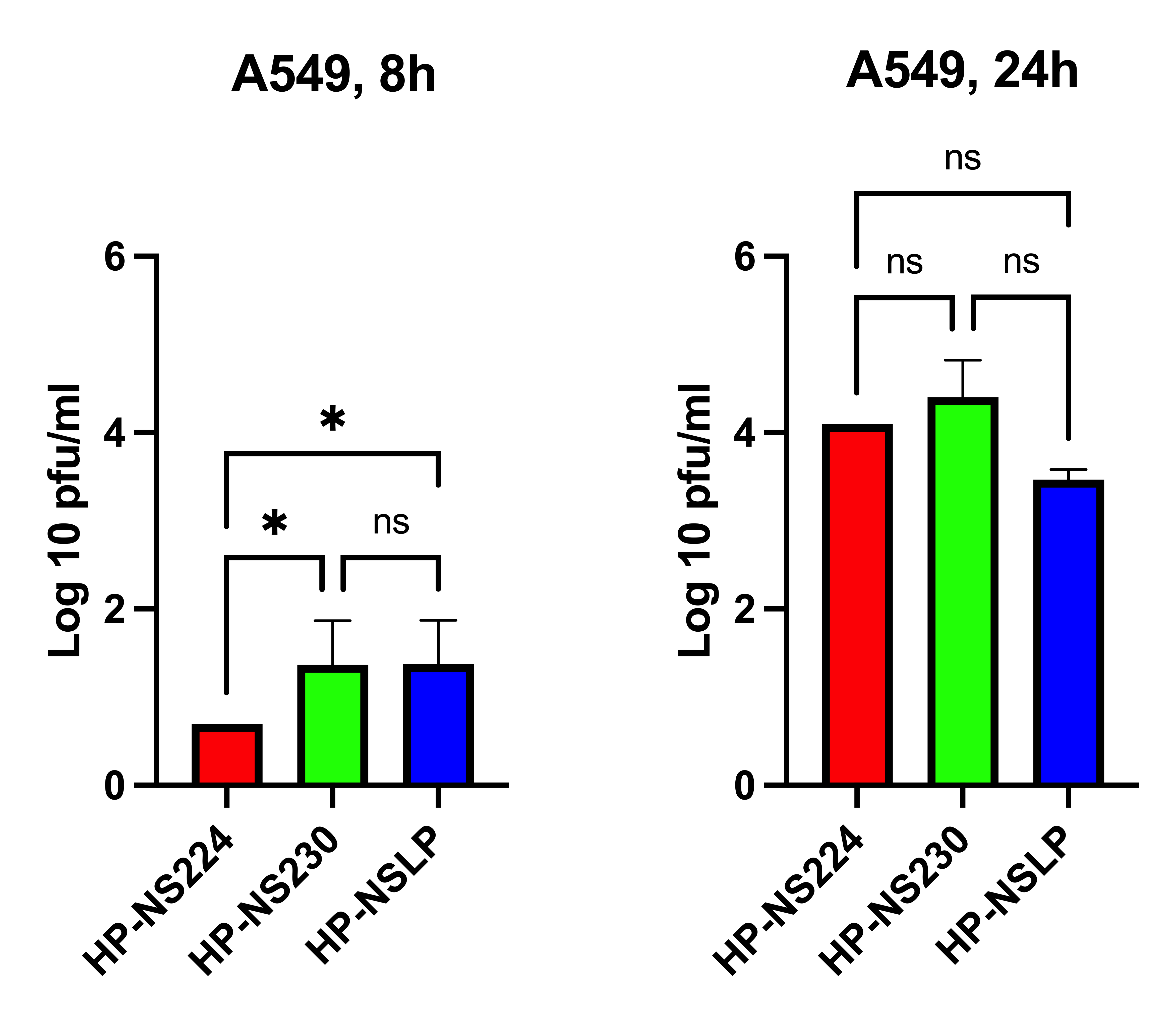

Supplement: Supplementary Figure S1.tiff [file KVIR_A_2379371_SM1285.tiff]
